# The chemokine receptor CXCR4 promotes granuloma formation by sustaining a mycobacteria-induced angiogenesis programme

**DOI:** 10.1038/srep45061

**Published:** 2017-03-23

**Authors:** Vincenzo Torraca, Claudia Tulotta, B. Ewa Snaar-Jagalska, Annemarie H. Meijer

**Affiliations:** 1 Institute of Biology, Leiden University, The Netherlands

## Abstract

CXC chemokine receptor 4 plays a critical role in chemotaxis and leukocyte differentiation. Furthermore, there is increasing evidence that links this receptor to angiogenesis. Using the well-established zebrafish-*Mycobacterium marinum* model for tuberculosis, angiogenesis was recently found to be important for the development of cellular aggregates called granulomas that contain the mycobacteria and are the hallmark of tuberculosis disease. Here, we found that initiation of the granuloma-associated proangiogenic programme requires CXCR4 signalling. The nascent granulomas in *cxcr4b*-deficient zebrafish embryos were poorly vascularised, which in turn also delayed bacterial growth. Suppressed infection expansion in *cxcr4b* mutants could not be attributed to an overall deficient recruitment of leukocytes or to different intramacrophage bacterial growth rate, as *cxcr4b* mutants displayed similar microbicidal capabilities against initial mycobacterial infection and the cellular composition of granulomatous lesions was similar to wildtype siblings. Expression of *vegfaa* was upregulated to a similar extent in *cxcr4b* mutants and wildtypes, suggesting that the granuloma vascularisation phenotype of *cxcr4b* mutants is independent of vascular endothelial growth factor.

CXCR4 is a critical chemokine receptor that controls migration and differentiation of a variety of cell types^1^,^2^. In the bone marrow, interaction of CXCR4 with its ligand CXCL12 (SDF1, stromal cell-derived factor 1) is required for retention of haematopoietic stem/progenitor cells and their complete differentiation before release into circulation^3^,^4^. During inflammatory responses, CXCR4 also sustains the trafficking of leukocytes to sites of inflammation^5^. Additionally, CXCR4 signalling on other cells is involved in key migratory mechanisms during development and has been linked to the metastatic behaviour of cancer cells^6^,^7^,^8^,^9^,^10^,^11^,^12^. CXCR4 signalling has also been connected to tumour-sustained angiogenesis^13^,^14^,^15^. In particular, CXCR4 stimulation was found to induce expression of vascular endothelial growth factor (VEGF) in human breast carcinoma cells and conversely blockade of CXCL12/CXCR4 signalling was able to suppress tumour angiogenesis and tumour growth *in vivo* in a murine model^13^. Finally, CXCR4 is also well known to play a critical function in HIV pathogenesis, since this factor represents an important HIV co-receptor, mediating viral entry into the host cells^16^.

The existence of a viable *cxcr4b* knockout has made the zebrafish model particularly attractive to characterise the function of CXCR4 signalling *in viv*o. Originally referred to as *Odysseus* or *Ody*, zebrafish *cxcr4b* mutants (*cxcr4b*^−/−^) were identified in a forward genetic screen, for factors involved in the migration of primordial germ cells^17^. The mutation harboured by *cxcr4b*^−/−^ consists in the conversion of the codon AAG in position 709–711 (coding the amino acid Lys_239_) into a stop codon, which determines the premature interruption of protein translation. The resulting truncated protein product lacks the third intracellular loop, the last two transmembrane domains, and the C-terminal domain, which compromises the 7-loop transmembrane architecture and therefore the capability of CXCR4 to couple with its ligand and its downstream partners (i.e. CXCL12 and heterotrimeric G-proteins)^17^. Similar to mammalian species, the zebrafish Cxcr4b/Cxcl12a signalling axis is also implicated in the retention and colonisation of haematopoietic tissues by haematopoietic stem cells, the recruitment of leukocytes to infectious foci and sites of injury, the invasiveness of cancer cells in tumour metastasis, and heart regeneration^4^,^12^,^18^,^19^,^20^.

Tuberculosis (TB) is caused by *Mycobacterium tuberculosis (Mtb*) infection and this disease typically manifests by the formation of aggregates of infected and non-infected immune cells that are known as granulomas. Several studies reported that human tuberculous granulomas, which frequently develop a hypoxic necrotising core, are extensively vascularised^21^,^22^,^23^. However, very little is understood about the actual relevance of angiogenesis for granuloma formation in TB patients. Additionally, the mechanism by which the vascularisation programme is initiated by the pathogen remains elusive^24^,^25^. In zebrafish larvae, *Mycobacterium marinum (Mm*), a close relative of *Mtb*, causes a disease that recapitulates significant aspects of human TB, which include the formation of necrotising granulomas and the initiation of specific transcriptional and morphological changes in *Mycobacterium*-infected macrophages^26^,^27^,^28^. Using the zebrafish model, it was recently found that *Mm* can also induce granuloma-associated angiogenesis and that initiation of this programme coincides with local induction of hypoxia and expression of the proangiogenic factor *vegfaa*^25^. Notably, the presence of macrophages was strictly necessary for mycobacterial-induced *vegfaa* expression and initiation of granuloma vascularisation. In tumours, activation of CXCR4/CXCL12 signalling is tightly linked to both the development of hypoxia and to the activation of angiogenesis^13^,^14^,^15^. Therefore, we hypothesised that this chemokine signalling axis could also be involved in granuloma-induced angiogenesis.

Here we show that Cxcr4b-deficient zebrafish larvae display an attenuated induction of the angiogenic programme at the nascent granulomas. Cellular composition of granulomas and chemotaxis of immune cells to infected areas were not altered in *cxcr4b* mutants compared to wt siblings. Additionally, *vegfaa* was still expressed in *cxcr4b* deficient larvae, despite the lack of granuloma vascularisation. Taken together, our study indicates that Cxcr4b-mediated signalling is required to mediate the full angiogenesis response to mycobacterial infections, and that suppression of pathological angiogenesis with CXCR4 blockers might represent an alternative therapeutic strategy to suppress granuloma-angiogenesis without perturbing VEGF signalling.

## Results

### Cxcr4b signalling controls granuloma-induced angiogenesis

**Figure 1:**
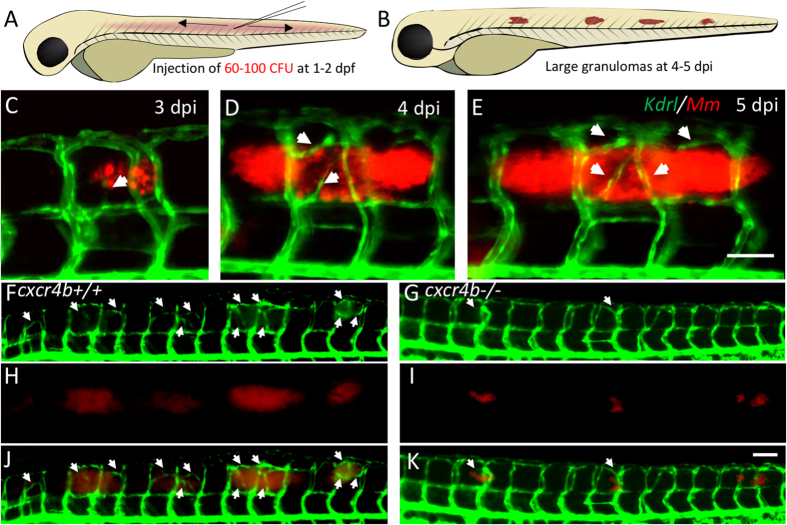
Trunk granuloma formation and induction of angiogenesis by *M. marinum*. (**A**,**B**) Schematic representation of injection location and of granuloma expansion in zebrafish embryos/larvae upon injection of *Mm* into the trunk. (**C**–**E**) Longitudinal imaging of vascular and bacterial growth during development of a trunk granuloma in the *Tg(kdrl:eGFP*) line at 3, 4 and 5 dpi. (**F**–**K**) Formation of trunk granulomas and their vascularisation in a *cxcr4b*^+/+^ and *cxcr4b*^−/−^ larva at 5 dpi. White arrows indicate sites of abnormal vascularisation associated to bacterial growth, notably largely initiated in the wt but rare in the mutant. See also Fig. 2 for quantification. Scale bars: 100 μm.

To study granuloma-associated angiogenesis, a trunk infection model was recently established in zebrafish embryos (Fig. 1A,B). In this model a transgenic *Tg(kdrl:eGFP*) background (labelling arterial and venous endothelium) was used to monitor host vascularisation in the environment of the nascent granulomatous lesions (Fig. 1C–E)^25^,^29^. To study whether *cxcr4b* has a function in granuloma vascularisation, we injected mCherry-fluorescent *Mm* in *cxcr4b* mutant and wildtype (wt) siblings at 2 days post fertilisation (dpf) and measured bacterial expansion and angiogenesis of the infected area at 5 days post infection (dpi) (Fig. 1F–K).

**Figure 2:**
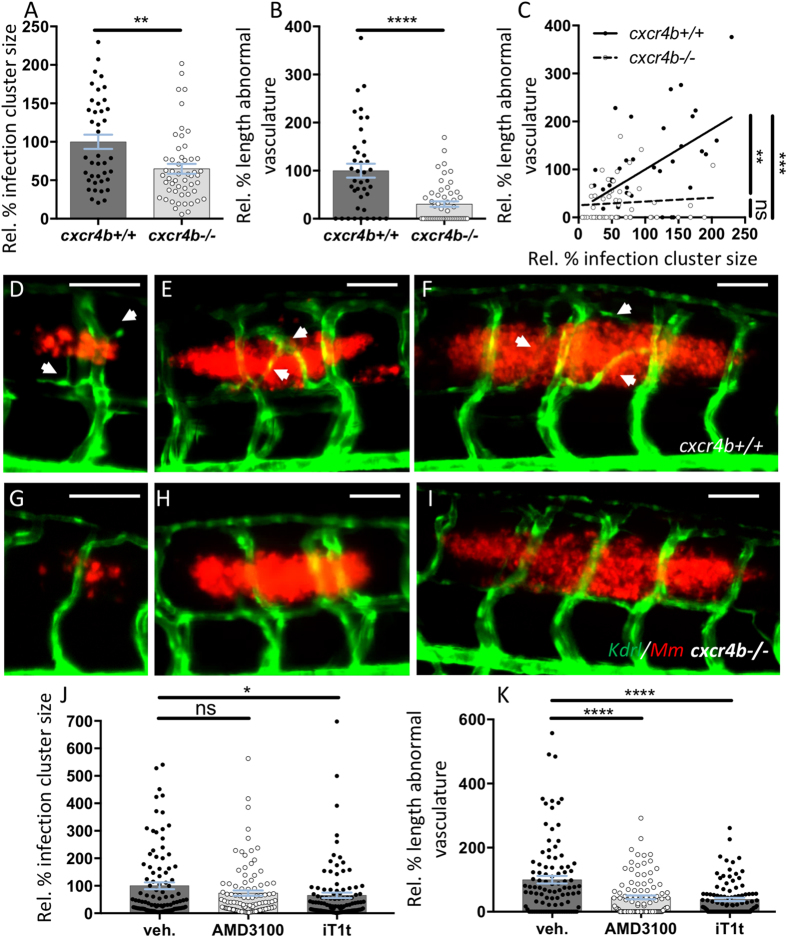
Cxcr4b mutation impairs granuloma-induced angiogenesis. (**A**,**B**) *cxcr4b* mutants display a reduced expansion rate of local granulomatous lesions (**A**), which coincided with the incapability to induce an angiogenic programme (**B**). (**C**) Vascularisation/granuloma expansion correlation analysis. In wt, the expansion of the granulomas depends on the activation of the angiogenic programme at the infection focus. Differently, in *cxcr4b* mutants, no significant correlation between granuloma size and vessel length could be observed and even large granulomas failed to initiate angiogenesis, suggesting that the differential activation of the angiogenic programme is not the effect, rather the cause, of reduced burden in mutants. Aberrant vasculature was measured as the two-dimensional length of vessels not found in uninfected larvae and that solely form in association to granulomas. Infection cluster size was measured as the two-dimensional area of individual trunk lesions. Values are expressed in percentage relative to average infected *cxcr4b*^+/+^ (set to 100%). Statistics in C indicates that the slopes of *cxcr4b*^−/−^ and *cxcr4b*^+/+^ trend lines are significantly different (linear regression comparison test) and that in wt (but not in mutants) this slope is different from 0 (*x* axis, Pearson correlation test). Each data point in A-C refers to individual trunk granulomas at 5 dpi. Experiments were performed in 3 replicates (cumulated in the graphs), each with 4–12 larvae per group. Total number of granulomas analysed: 40 (*cxcr4b*^+/+^), 52 (*cxcr4b*^−/−^). (**D**–**I**) Representative images of comparably-sized 5 dpi trunk granulomas in *cxcr4b*^−/−^ and *cxcr4b*^+/+^ (quantified in **A–C**). Notably *cxcr4b*^−/−^ granulomas display a defect in local vascularisation, which is independent of the granuloma size. Scale bars: 100 μm. (**J**–**K**) Treatments with CXCR4 antagonists AMD3100 and IT1t phenocopy *cxcr4b* mutants and display attenuated (IT1t) expansion of granulomatous lesions (**J**) and reduced (AMD3100 and IT1t) induction of granuloma-associated angiogenesis (**K**). Experiments in **J–K** were performed in *Tg(fli1a:eGFP*) background. Data are analysed as in Fig. 2A,B. Experiments were performed in 2 replicates (cumulated in the graphs). Total number of granulomas analysed: 101 (vehicle), 101 (AMD3100), 103 (IT1t).

In *cxcr4b*^−/−^ larvae, the expansion of the infection progressed at a lower rate as compared to the lesions in wt (Fig. 1F–K). Simultaneously, the association of angiogenesis with the granulomas was impaired in these mutants and, differently from wt, *cxcr4b* mutants did not have a significant positive correlation between granuloma size and length of associated abnormal vasculature (Fig. 2A–C). Indeed, large granulomas failed to induce angiogenesis in *cxcr4b*^−/−^ (Fig. 2D–I). The interdependence between granuloma expansion and angiogenesis had been previously described^25^ and mechanistically closely resembles the angiogenic switch in tumorigenesis, in which tumour size is directly related to the local induction of pathogenic angiogenesis^30^. Altogether, our findings suggest that *cxcr4b* mutation affects granuloma expansion by primarily affecting the initiation of the angiogenesis programme, and the difference in infection burden appears to be the consequence, rather than the cause, of impaired angiogenic support of granuloma formation. Notably, pharmacological inhibition of Cxcr4b signalling by application of two CXCR4 antagonists, namely AMD3100 and IT1t, recapitulated the angiogenesis phenotype of *cxcr4b* genetic knockouts with significant reduction of abnormal vasculature associated to granulomas. The strongest effect was observed in case of IT1t application, which also reduced granuloma size, highlighting the therapeutic potential of CXCR4 blockers to counteract mycobacterial diseases (Fig. 2J,K).

### *cxcr4b* mutation does not alter the migratory and microbicidal capabilities of macrophages or the cellular composition of the granulomatous lesions

**Figure 3:**
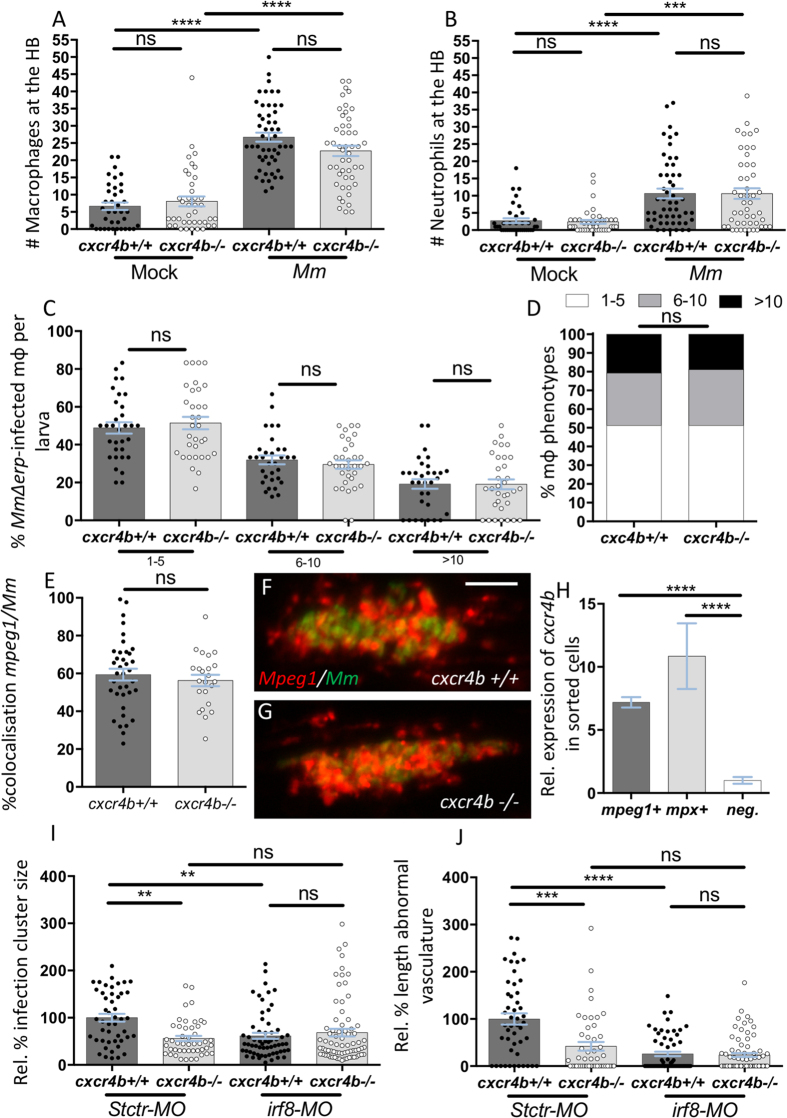
c*xcr4b* is not required for the establishment of macrophage parasitism, but macrophages are necessary to mediate *cxcr4b*-dependent angiogenesis. (**A**,**B**) Recruitment of macrophages (**A**) and neutrophils (**B**) 3 hours post local hindbrain (HB) infection at 2 dpf with *Mm* was unaffected by *cxcr4b* mutation. Each data point in (**A**,**B**) represents the recruitment measured in 1 embryo. Experiments were performed in 2 replicates (cumulated in the graphs), each with 17–35 embryos per group. Total number of embryos analysed: 38 (Mock*-cxcr4b*^+/+^), 41 (Mock*-cxcr4b*^−/−^), 53 (*Mm-cxcr4b*^+/+^), 49 (*Mm-cxcr4b*^−/−^). **C–D** *cxcr4b* is dispensable for the capability of macrophages to counteract intracellular replication of *Mm Δerp.* Infected macrophages (*mpeg1:mCherry-F*^+^) were classified into three phenotypic classes, according to the severity of intracellular infection (1–5, 6–10, or >10 bacteria). (**C**) represents the percentage of macrophages per larva that populates each class. (**D**) represents the overall distribution of macrophage phenotypes (macrophages from all larvae cumulated). Experiments were performed in 2 replicates (cumulated in the graphs), each with 16–17 larvae per group. Total number of larvae analysed in C: 32 (*cxcr4b*^+/+^), 33 (*cxcr4b*^−/−^). Total number of macrophages analysed in D: 387 (*cxcr4b*^+/+^), 334 (*cxcr4b*^−/−^). (**E**–**G**) % of *mpeg1:mCherry-F* signal overlapping with *Mm-GFP* signal in *cxcr4b*^−/−^ and *cxcr4b*^+/+^. No significant deviation in macrophage composition of the trunk granuloma lesions was detected. Each data point represents 1 granuloma. Experiment was performed in 1 replicate. Total number of granuloma analysed: 37 (*cxcr4b*^+/+^), 23 (*cxcr4b*^−/−^). Representative example images are shown in (**F** and **G**). (**H**) Expression of *cxcr4b* in macrophages (*mpeg1*^+^) and neutrophils (*mpx*^+^). Both cell subsets express *cxcr4b* at significantly higher levels than the negative (non-fluorescent) cell population. Data represent fold changes relative to the negative cell fraction. Cells were sorted at 2 dpf, from at least 100 embryos. Experiment was performed in 3 replicates. I-J. Macrophages are indispensable to mediate granuloma vascularisation (**I**) and an increased number of neutrophils (by *irf8* morpholino knockdown) cannot compensate for macrophage deficiency, neither in *cxcr4b*^−/−^ nor in *cxcr4b*^+/+^. Deficient bacterial expansion in *irf8* knockdown condition (*irf8-MO*) compared to standard control morpholino treatment (*Stctr-MO*) can be attributed to the lack of vascularisation as suggested by non-significant differences in burden between the knockdowns and control *cxcr4b*^−/−^ (J). Data are analysed as in Fig. 2A,B, but infections were performed at 33 hpf, instead of 2 dpf. Experiments were performed in 1 replicate. Total number of granuloma analysed: 47 (*Stctr-MO-cxcr4b*^+/+^), 47 (*Stctr-MO-cxcr4b*^−/−^), 61 (*irf8-MO-cxcr4b*^+/+^), 73 (*irf8-MO-cxcr4b*^−/−^).

Intramacrophage residence of mycobacteria is indispensable for initiation of angiogenesis^25^. Therefore, we investigated whether the difference in promotion of angiogenesis in *cxcr4b* mutants could be explained by aberrant macrophage recruitment (resulting in different intracellular/extracellular ratios of *Mm*), differential microbicidal capability of macrophages, or alteration in the macrophage composition of granulomatous lesions. When mycobacteria were injected locally into the hindbrain ventricle, a comparable number of leukocytes were promptly recruited (3 hpi) to the infected site in mutants and wt (Fig. 3A,B). To assess the possibility of an altered microbicidal capability, we injected the *Mm* mutant strain *Δerp*, which is highly susceptible to macrophage clearance and replicates intracellularly at a low rate, thereby permitting quantification of mycobacterial growth in individual macrophages by live microscopy. At 44 hpi the percentage of macrophages displaying low (1–5 bacteria), moderate (6–10 bacteria) or high (>10 bacteria) infection load was quantified and *cxcr4b* mutants showed similar distribution of the infection phenotypes as wt siblings, indicating that depletion of *cxcr4b* does not alter the macrophage capability to counteract intracellular infection (Fig. 3C,D). We also excluded that *cxcr4b* mutation might affect the content of macrophages in the mature granulomas, as the percentage of mycobacteria co-localising with macrophages was similar in mutant and wt siblings (Fig. 3E–G). In zebrafish larvae, *cxcr4b* is expressed both by the macrophage lineage (marked by *mpeg1*) and by the neutrophil lineage (marked by *mpx*) (Fig. 3H). However, it is unlikely that the angiogenesis deficiency in *cxcr4b* mutants depended on *cxcr4b* expression by neutrophils, as suggested by *irf8* morpholino knockdown, which skews myelopoiesis towards neutropoiesis at the expense of primitive macrophage development (Fig. 3I,J)^31^. In this situation the initial deficiency in macrophages was already sufficient to abrogate the angiogenic response, indicating that neutrophils alone cannot support angiogenesis and therefore that the *cxcr4b* mutation phenotype implicates a macrophage-related function. However, since recruitment of macrophages, cellular composition of lesions and intramacrophage killing of bacteria were comparable between mutants and wt (Fig. 3), we hypothesised that *cxcr4b* mainly affects the interaction of the macrophages with the surrounding tissue.

### Mutation of *cxcr4b* does not interfere with induction of Vegf signalling and with physiological angiogenesis

**Figure 4:**
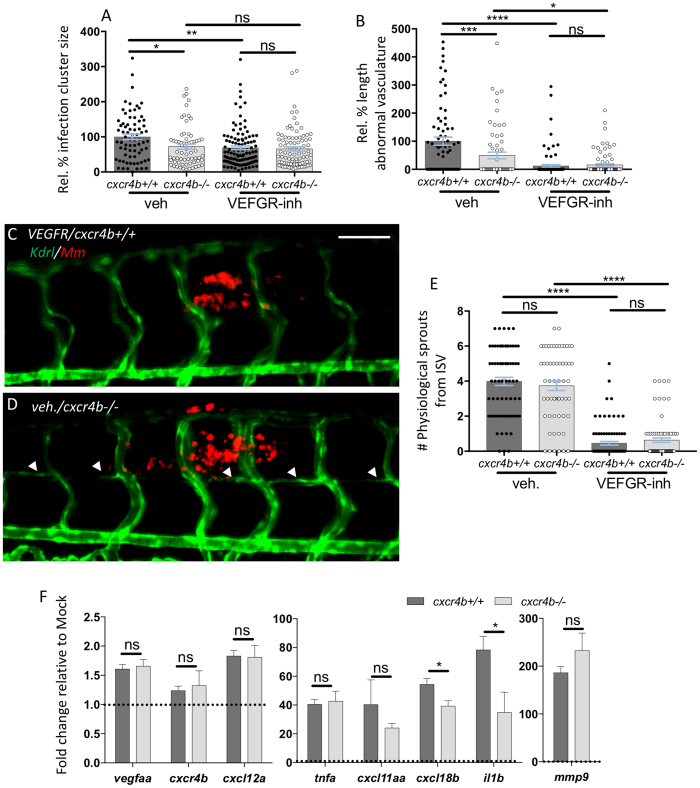
Deficient granuloma vascularisation in *cxcr4b* mutation does not strictly depend on aberrant Vegf signalling or attenuated expression of inflammatory genes. (**A**,**B**) *cxcr4b* deficiency reduced granuloma expansion to a similar extent as blockade of Vegf signalling (VEGFR-inh, treatment with Sunitinib) (**A**), although Vegf inhibition can more severely affect angiogenesis. (**B**). Data are analysed as in Fig. 2A,B. Experiments were performed in 2 replicates (cumulated in the graphs). Total number of granulomas analysed: 73 (vehicle*-cxcr4b*^+/+^), 66 (vehicle*-cxcr4b*^−/−^), 109 (VEGFR-inhibitor*-cxcr4b*^+/+^), 88 (VEGFR-inhibitor*-cxcr4b*^−/−^). **C–E** Inhibition of Vegf signalling (**C**), but not depletion of *cxcr4b* (**D**), severely affects physiological angiogenesis. Quantification in E was performed by counting the number of sprouts from intersegmental vessels (ISV) from images encompassing 6–7 somites in the trunk region (as represented in **C**,**D**). Experiment was performed in 2 replicates (cumulated in the graph). Total number of region of interest analysed: 73 (vehicle*-cxcr4b*^+/+^), 59 (vehicle*-cxcr4b*^−/−^), 93 (VEGFR-inhibitor*-cxcr4b*^+/+^), 77 (VEGFR-inhibitor*-cxcr4b*^−/−^). Scale bar: 100 μm. (**F**) At 5 dpi, Cxcr4b does not exert a transcriptional control on *vegfaa* expression, but affects the inflammatory response to mycobacterial infection, in particular by dampening *il1b* expression. qRT-PCR were performed whole mount from pools of at least 10 embryos (4 replicates). Data represent fold changes of *Mm* infection groups relative to their mock injection (2% PVP in PBS) control group (set to 1, dotted line). No significant differences in basal expression levels were found between mutants and wt for the analysed genes.

Since CXCR4 was previously linked to tumour angiogenesis via a transcriptional control on VEGF signalling^13^, we addressed whether *cxcr4b* mutation controlled granuloma angiogenesis by affecting Vegf signalling. Treatment with Sunitinib (a Vegf receptor inhibitor)^32^ or *cxcr4b* mutation reduced trunk granuloma size to the same level (Fig. 4A), although Vegf receptor inhibition could still synergise with *cxcr4b* mutation and could suppress more severely granuloma vascularisation (Fig. 4B). However, it should be noted that treatment with Sunitinib also affected to some extent physiological angiogenesis, as the frequency of physiological sprouting from intersegmental vessels (ISVs) and their fusion to the following ISV was also reduced by this treatment (Fig. 4C–E). In contrast, *cxcr4b* mutation affected specifically the granuloma-induced angiogenesis and not the physiological sprouting of axial vessels from ISVs. Since the angiogenesis response to mycobacterial infection was found to coincide with local induction of *vegfaa*^25^ and mammalian CXCR4 has been linked to a transcriptional regulation of *VEGFA*^13^, we addressed whether *cxcr4b* affected granuloma-induced angiogenesis by exerting a similar transcriptional control on *vegfaa* expression in our model. Whole mount qRT-PCR analysis revealed that both mutants and wt upregulated *vegfaa* to a comparable level (Fig. 4F). We also found that the levels of *vegfaa* induction, although significant, were limited (1.5-fold) when compared to the induction of other infection-inducible genes, which could be seen highly upregulated in the same conditions (Fig. 4F). Likewise, expression of *cxcl12a* (encoding the ligand of Cxcr4b) showed limited but comparable induction in mutants and wt (Fig. 4F). Expression of *cxcr4b* mRNA was not relevantly altered by the infection and remained comparable between *wt* and mutants (as mentioned above, the *cxcr4b* mutant mRNA differs from the wt only because of a non-sense point mutation and can, therefore, be normally tested by qRT-PCR). These results suggest that the reduced angiogenic response to granulomas in *cxcr4b* mutants is not dependent on Vegf signalling, and that Cxcr4b axis might therefore act downstream of this pathway.

### Cxcr4b mutation is associated with reduced inflammatory gene expression but *il1b* signalling is dispensable for granuloma angiogenesis

Signalling via CXCR4 has been linked to the induction of inflammatory genes during the response to infections^33^,^34^ and the local chronic induction of inflammation mediators is known to play a critical role to sustain angiogenesis of damaged/inflamed tissues^35^,^36^. Several inflammatory molecules (such as IL1β and TNFα) have been largely connected to the sustenance of pathological angiogenesis in inflammatory diseases^35^,^37^. In agreement, the use of the transgenic reporter *Tg(il1b:eGFP-F*)^38^ revealed high expression of *il1b* by *Mm*-infected cells composing the granulomatous lesions in zebrafish larvae (Supplementary Figure S1). Therefore, we analysed the expression profile of *il1b* and several other *Mm*-inducible inflammatory genes (*tnfa, cxcl11aa, cxcl18b* and *mmp9*)^39^,^40^,^41^. All these genes could be still induced in *cxcr4b* mutants. However, two of the analysed genes, namely *cxcl18b* and *il1b* were significantly less induced in the mutants, when compared to wt (Fig. 4F). This suggests that a differential inflammatory response can be elicited in *cxcr4b* mutants and wt during the infection progression. To address whether the differential induction of *il1b* might play a role in mediating the *cxcr4b* phenotype, we depleted *il1b* by morpholino knockdown. Morphants displayed comparable vascularisation of trunk granulomas as controls, therefore excluding that the *cxcr4b* mutant phenotype could be caused by reduced *il1b* signalling (Supplementary Figure S1).

In conclusion, our data show that Cxcr4b is required for granuloma vascularisation by a mechanism that appears to be independent from Vegf or the primary proinflammatory cytokine Il1β. Loss of Cxcr4b function limits granuloma expansion to a similar degree as Vegfr blockade, suggesting CXCR4 inhibition as an alternative host-directed therapeutic approach for TB treatment without side effects on physiological angiogenesis.

## Discussion

Using intravital imaging in the zebrafish–*Mm* infection model, we have investigated the function of the homoeostatic chemokine receptor Cxcr4b in the development of mycobacterial infection and granuloma formation. We found that zebrafish embryos/larvae carrying a homozygote mutation of *cxcr4b,* developed an attenuated disease which could be associated with the inability of these mutants to induce local angiogenesis. This granuloma angiogenesis defect could be recapitulated with pharmacological inhibitors of CXCR4 (AMD3100 and IT1t). Supporting that Cxcr4b promotes granuloma formation by sustaining a mycobacteria-induced angiogenesis programme, the positive correlation between granuloma growth and angiogenesis that is normally observed in wt larvae, was lost in *cxcr4b* mutants. Furthermore, pharmacological blockade of the angiogenesis programme by Vegfr inhibitor could fully abolish the differences between mutants and wt in the exacerbation of mycobacterial infection. The use of angiogenesis inhibitors has previously been proposed as a host-targeted therapy to suppress the formation of granulomas in TB patients^25^. Based on our results, the CXCR4 receptor could be explored as a novel target for anti-angiogenic tuberculosis therapy.

It is unlikely that the granuloma angiogenesis phenotype requires expression of *cxcr4b* by the endothelial cells *per se*, since several studies have identified that venous/arterial endothelium (*kdrl*^+^) does not express high levels of *cxcr4b* and that this gene can be found expressed only in developing lymphatic vessels (*kdrl*^−^)^18^,^42^. Our study suggests that *cxcr4b* function in evoking angiogenesis requires the presence of macrophages, since skewing haematopoiesis towards neutrophils at the expenses of macrophages severely affected the induction of the granuloma vascularisation. This conclusion is in line with previous data showing that macrophage depletion by transcription factor *spi1*/*pu.1* knockdown reduced the formation of new vessels to the *Mm* infection foci^25^. Together, these results support that *cxcr4b* exerts its function on macrophages in a cell-autonomous fashion and affects their capability to promote angiogenesis.

Macrophages of *cxcr4b* mutants were normally capable of migrating to initial mycobacterial infection foci and were equally suited to contain initial intramacrophage bacterial replication, therefore excluding significant effects of *cxcr4b* on the establishment of mycobacteria/macrophage parasitism. Apart from the deficiency in the expansion rate and the absence of associated angiogenesis, infectious lesions of *cxcr4b*^−/−^ were structurally undistinguishable from those of wt when granulomas of similar sizes were compared. Since previous studies reported that the *vegfaa* expression pattern coincided with the local promotion of granuloma angiogenesis^25^, we hypothesised that Cxcr4b might support angiogenesis via promotion of *vegfaa* expression. The induction levels of *vegfaa* in *Mm* infected larvae were low and we could not detect a significant difference between infected *cxcr4b* mutants and wt. Several studies have shown that VEGF induction in inflammatory processes can be transient and largely variable^43^,^44^,^45^. Therefore, a direct function of *cxcr4b* in the regulation of Vegf/Vegfr signalling cannot be completely excluded by our study. Interestingly, we observed a pronounced effect of Vegfr-inhibition, but not *cxcr4b* mutation, on the formation of physiological vessel sprouts from the intersegmental vessels, indicating that while Vegfr-inhibitors act as wide-spectrum angiogenesis blockers, *cxcr4b* depletion solely inhibits granuloma-induced angiogenesis.

Notably, *cxcr4b* mutation affected propagation of the inflammatory response, since expression of the master inflammatory gene *il1b* was significantly attenuated in *cxcr4b* mutants. Several reports showed that CXCR4 signalling can support inflammation. In particular, elegant studies revealed that CXCR4 can cross-talk with the plasma membrane Toll-like receptor TLR4 and act as a potent co-stimulatory mediator^33^,^34^. There is also growing evidence of a tight link between the angiogenesis process and inflammation, since many inflammatory molecules (prostaglandins, TNFα, interleukins such as Il1β) are proangiogenic too^35^,^36^,^37^,^46^,^47^. Therefore, we investigated whether curtailing inflammation via *il1b* depletion could phenocopy *cxcr4b* mutation in granuloma-angiogenesis. However, our data indicate that suppression of IL1β *per se* is not sufficient to abrogate the angiogenic response to the granulomas.

The relevance of inflammation in triggering granuloma-associated angiogenesis and the exact pathways by which Cxcr4b controls the proangiogenic signalling awaits a more detailed characterisation and the zebrafish model may represent an elective surrogate system to further address the angiogenesis/inflammation interplay *in vivo*. An attractive hypothesis is that CXCR4 signalling might control the function that macrophages exert in facilitating the engagement of endothelial cells from pre-existing vessels. Recently, macrophages have been shown to promote vascular anastomosis, a process by which neighbouring endothelial cells are connected to form new vessels, occurring in angiogenesis and *de novo* vasculogenesis^48^. Notably, this report also suggests that macrophage-mediated anastomosis acts downstream of Vegf-mediated tip cells induction, delineating a two-step model compatible with our finding that *cxcr4b* mutants are unable to complete the angiogenic programme, despite the fact that Vegfaa signalling is induced.

Concluding, the angiogenesis programme mounted at the granuloma is important to sustain the further expansion of the lesion and is a complex multifaceted process that involves pathogen virulence factors, macrophage response to intracellular infection and granuloma aggregation, induction of local hypoxia and VEGF signalling^25^. Our addition to this scenario is that the homoeostatic chemokine receptor CXCR4 is also critical to permit the induction of the angiogenic programme and to contain the propagation of inflammation. While inhibition of Vegf signalling or depletion of *cxcr4b* lead to comparable attenuation of granuloma expansion in the zebrafish model, blockade of Vegf signalling significantly affected also physiological vascularisation, while the effect of *cxcr4b* mutation was specific towards the granuloma-associated vasculature. In terms of therapeutic applications, this suggests that antagonising CXCR4 signalling might be a preferred system to specifically curtail pathological angiogenesis. Additionally, in case of TB infections, blockade of CXCR4 may represent a preferential treatment of HIV/*Mtb* co-infected patients. Given the fact that CXCR4 plays a relevant function in HIV entry, suppression of CXCR4 could simultaneously counteract both mycobacterial and viral infection.

## Methods

### Zebrafish lines and maintenance

Zebrafish lines were handled in compliance with the local animal welfare regulations and maintained according to standard protocols (zfin.org). The study was approved by the local animal welfare committee (DEC) of the University of Leiden (licence number: 10612, protocol 14227). Fish lines used in this work were the following: wildtype strain AB/TL, *Tg(il1b:eGFP-F*^*zf550*^)^38^, *Tg(fli1a:eGFP*^*y1*^)^49^, the double transgenic line *Tg(mpeg1:mCherry-F*^*ump2*^/*mpx:eGFP*^*i114*^)^50^,^51^, *cxcr4b* homozygote mutant (*cxcr4b*^−/−^) and wildtype (*cxcr4b*^+/+^) siblings of (*cxcr4b*^*t26035*^)^12^,^17^ crossed into the transgenic backgrounds of *Tg(kdrl:eGFP*^*s843*^)^29^ or *Tg(mpeg1:mCherry-F*^*ump2*^). Embryos were grown at 28.5 °C in egg water (60 μg/ml sea salt, Sera Marin, Heinsberg, Germany). Larvae destined to image acquisition were maintained in egg water supplemented with 0.003% PTU (1-phenyl-2-thiourea, Sigma-Aldrich, St Louis, MO, USA) from 8–12 hpf to prevent melanisation. Anaesthesia of embryos/larvae used for live imaging was achieved with 0.02% buffered Tricaine (3-aminobenzoic acid ethyl ester, Sigma-Aldrich) in egg water.

### Bacterial cultures and infection delivery

Approximately 60–100 CFU suspended in a volume of 10 nl of *M. marinum* strain M (or where specified its isogenic mutant strain *Δerp*) constitutively expressing mCherry, eGFP or mWasabi^52^,^53^ were injected into the trunk at 2 dpf^25^, while approximately 50 CFU in a volume of 1 nl were injected into the hindbrain (HB, 2 dpf). For recruitment assays and qRT-PCR experiments, the same volume of mock [2% polyvinylpyrrolidone-40 (Sigma-Aldrich) in phosphate buffer saline (PBS)] was injected in control embryos, as a reference control. Bacteria were grown and harvested from an O/N culture as described previously^40^,^54^. For morpholino experiments (*irf8* and *il1b* knockdown) and collection of samples for qRT-PCR, trunk infections were performed at 33 hpf (instead of 2 dpf). For the assessment of the microbicidal activity of macrophages against initial mycobacterial infection, single cell suspensions of *Mm Δerp*-mWasabi were injected into the caudal vein at 33 hpf from −80 °C frozen single-use aliquots, using a protocol adapted from reference^53^. Briefly, bacteria from a 1-week-old plate were inoculated to an OD600 of 0.2 in 10 ml 7H9 medium supplemented with ADC enrichment. The culture was grown for 24 h to reach an OD of approximately 1.0. Bacteria were washed 3 times with PBS and suspended in PBS supplemented with 10% glycerol to an OD600 of 5.0. To generate a single cell suspension, bacteria were passed 10 times through a syringe. 50 μl aliquots were frozen in liquid nitrogen and stored at −80 °C. Upon thawing, the vital bacteria were quantified by plating as being approximately 100 CFU/nl. For quantification of macrophage and neutrophils recruitment to mycobacteria, 2 dpf embryos were infected in the hindbrain, fixed at 3 hpi in 4% paraformaldehyde in PBS supplemented with 0.08% Triton X-100 and prepared for combined L-plastin (Lp) immunostaining and myeloperoxidase (Mpx) enzymatic activity staining as in reference^55^. Leukocytes accumulated at the injected cavity (macrophages: Lp-positive and Mpx-negative; neutrophils: Mpx-positive) were counted using a Leica MZ16FA fluorescence stereomicroscope (Leica Microsystems, Rijswijk, The Netherlands).

### RNA isolation, FACS and qRT-PCR

To evaluate the induction of genes upon infections (Fig. 4F), whole-embryo RNA extraction, cDNA synthesis and qRT-PCR were performed at 5 dpi from a pool of embryos injected at 33 hpi (4 replicates), according to the procedure described in reference^40^. To demonstrate expression by phagocytes (Fig. 3H), RNA was isolated from *mpx*^+^ and *mpeg1*^+^ cells sorted at 2 dpf from the double transgenic line *Tg(mpeg1:mCherry-F*^*ump2*^/*mpx:eGFP*^*i114*^), according to previous reports^40^,^56^. Reference housekeeping genes were *ppia1b* for whole-mount samples and *eif4a1b* for FACS-sorted samples. qRT-PCR primers are reported in Supplementary Table S1.

### Pharmacological inhibition of VEGF and CXCR4 signalling

For pharmacological inhibition of VEGF signalling, the VEGFR tyrosine kinase inhibitor Sunitinib (1 μM, Sigma-Aldrich) or vehicle treatment (0.1% DMSO, Sigma-Aldrich), were applied directly to the egg water (immediately after infection) and refreshed every 2 days according to reference^32^. Similarly, for pharmacological suppression of the CXCR4 axis, we applied the CXCR4 antagonists AMD3100 (25 μM, Sigma-Aldrich)^18^, IT1t (20 μM, Calbiochem, Merk, Darmstadt, Germany)^12^ or vehicle treatment (0.1% DMSO) via bath exposure of infected embryos. In this case drugs were not refreshed during the whole experimental course.

### *Irf8* and *il1b* knockdown

All morpholinos were obtained from Gene tools. 1 nl of 1 mM *irf8* splicing morpholino (5′-AATGTTTCGCTTACTTTGAAAATGG-3′)^31^, 1 nl of 0.6 mM *il1b* splicing morpholino (5′-CCCACAAACTGCAAAATATCAGCTT-3′)^57^,^58^, or the same concentration and volumes of a standard control morpholino (5′-CCTCTTACCTCAGTTACAATTTATA-3′) were injected in one-cell stage zebrafish fertilised eggs according to reference^31^.

### Imaging and image quantification

Fixed or live embryos and larvae were imaged using a Leica MZ16FA fluorescence stereomicroscope. The size of individual granulomas was quantified at 5 dpi (at 4 dpi in case of *irf8* or *il1b* morpholino knockdown) by pixel count, using Fiji/ImageJ software (NIH, Bethesda, MD, USA). The length of abnormal vasculature at the granulomas was quantified at the same stage from images according to reference^25^ and using the *Tg(kdrl:eGFP*) or, where specified, the *Tg(fli1a:eGFP*) transgenic lines. Briefly, the cumulated two-dimensional length of vessels not seen in uninfected larvae (aberrant sprouts from the dorsal longitudinal anastomic vessel or from the intersegmental vessels, and axial vessels anomalously redirected to grow towards the granulomas) was measured per each granuloma. To score the microbicidal activity of macrophages against mycobacteria, *MmΔerp*-mWasabi bacteria prepared as described above were injected into the caudal vein of *Tg(mpeg1:mCherry-F*)/*cxcr4b*^−/−^ and *Tg(mpeg1:mCherry-F*)/*cxcr4b*^+/+^ embryos. Intramacrophage mycobacterial sites of growth were counted (blind) from fixed embryos at 44 hpi according to reference^53^, using Zeiss Observer 6.5.32 confocal microscope and a C-Apochromat 63x/1.20 W Korr UV-VIR-IR M27 objective (Carl Zeiss, Sliedrecht, The Netherlands). The level of infection per macrophage was classified into three groups based on bacterial content (1–5 bacteria, 6–10 bacteria or >10 bacteria). To estimate similar macrophage content of granulomas, the percentage of colocalisation of *Tg(mpeg1:mCherry-F*) and *Mm* M-eGFP was quantified ad 5 dpi using Fiji/ImageJ dedicated colocalisation plugin. Confocal images in Supplementary Figure S1 were taken from trunk granulomas at 5 dpi/6 dpf, using Zeiss Observer 6.5.32 confocal microscope and an EC Plan-Neofluar 20x/0.50 M27 (Supplementary Figure S1A) or C-Apochromat 63x/1.20W Korr UV-VIR-IR M27 objective (Supplementary Figure S1B).

### Statistical analysis

Statistical significance was analysed using GraphPad Prism 6 or 7 (GraphPad Software, La Jolla, CA, USA). Differences in granuloma sizes, angiogenesis, and bacteria/macrophage co-localisation (Fig. 2A–B–J–K, Fig. 3E–I–J, Fig. 4A,B, Supplementary Figure S1C,D) were statistically tested by unpaired *t-*test (comparison between 2 groups) or one-way ANOVA followed by Šidák comparison test (multiple group comparisons). Difference in number of leukocytes recruited and number of physiological sprouts (Fig. 4A–B–E) were analysed by Kruskal-Wallis test for non-parametric data followed by Šidák comparison test. Bacterial cluster size/angiogenesis correlation (Fig. 2C) was analysed by Pearson correlation test and the difference between mutant and wt regressions was computed by GraphPad Prism dedicated linear regression analysis tool. Individual unpaired *t*-tests per each class of macrophage phenotype were used to compare the percentage of *MmΔerp*-infected macrophages per larva (Fig. 3C), while a chi-square contingency test was used to test the overall distribution of macrophage phenotypes (Fig. 3D).

For qRT-PCR (Fig. 4F), statistical significance was estimated by two-tailed *t*-tests on ln(n)-transformed relative induction folds. Significance (*P*-value) is indicated with: ns, non-significant; *P < 0.05; **P < 0.01; ***P < 0.001, ****P < 0.0001. Error bars: mean ± s.e.m.

## Additional Information

**How to cite this article**: Torraca, V. *et al*. The chemokine receptor CXCR4 promotes granuloma formation by sustaining a mycobacteria-induced angiogenesis programme. *Sci. Rep.* **7**, 45061; doi: 10.1038/srep45061 (2017).

**Publisher's note:** Springer Nature remains neutral with regard to jurisdictional claims in published maps and institutional affiliations.

## Supplementary Material

Supplementary Information
