# Supplementary material for: The chemokine receptor CXCR4 promotes granuloma formation by sustaining a mycobacteria-induced angiogenesis programme
Source: Sci Rep. 2017 Mar 23;7:45061. doi: 10.1038/srep45061 (PMC5362882; doi:10.1038/srep45061)
Supplement: Supplementary Information [file srep45061-s1.pdf]

The chemokine receptor CXCR4 promotes granuloma formation by sustaining a mycobacteria-induced angiogenesis programme

Vincenzo Torraca, Claudia Tulotta, B. Ewa Snaar-Jagalska and Annemarie H. Meijer*

Affiliation

Institute of Biology, Leiden University, The Netherlands

* Corresponding Author:

prof. dr. Annemarie H. Meijer

e-mail: a.h.meijer@biology.leidenuniv.nl

address: Institute of Biology, Leiden University, Einsteinweg 55, 2333 CC, Leiden, The Netherlands

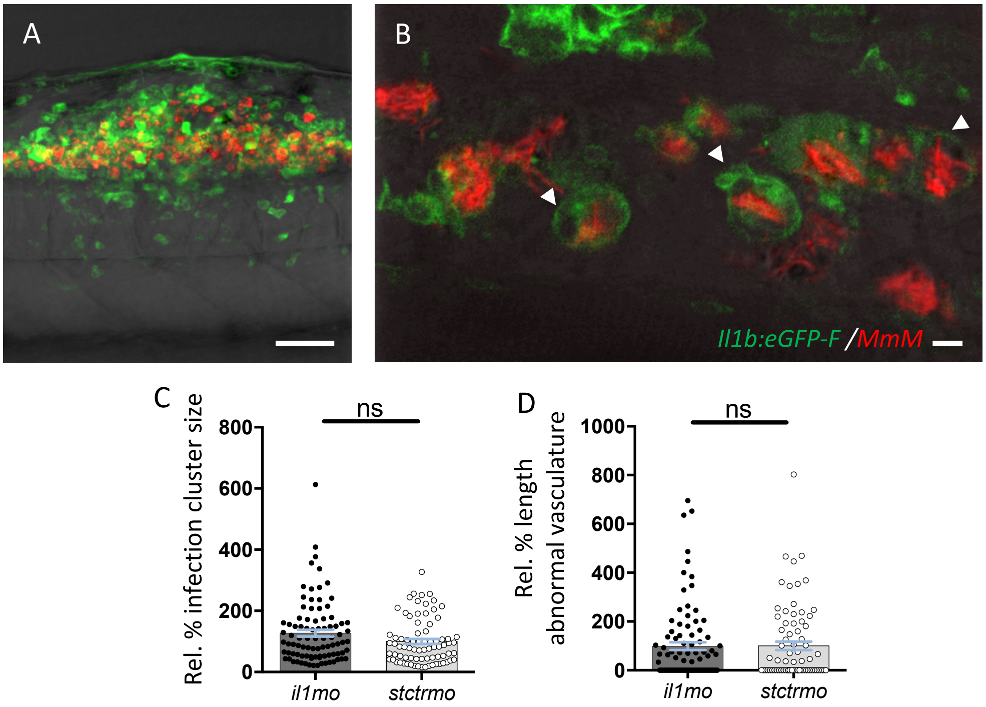

Supplementary Figure S1

Supplementary Figure S1. Function of *il1b* on trunk granulomas. A-B. Induction of the proangiogenetic inflammatory mediator *il1b* at the trunk granuloma. The large granuloma forming in the trunk contains a high number of *il1b*-expressing cells, predominantly consisting of infected cells. Confocal images were acquired at 5 dpi from infected *Tg(il1b:eGFP-F)* larvae. Scale bars: A: 50 µm; B: 5 µm. **C-D.** Despite being largely induced in infected cells during trunk granuloma formation, and notably suppressed by *cxcr4b* mutation, depletion of *il1b* does not recapitulate *cxcr4b* phenotype in granuloma vascularisation, since il1b morphants could still develop normally vascularise granulomas. Data were obtained and analysed as in Figure 3I-J (2 replicates, cumulated).

Supplementary Table S1. Primers used for qRT-PCR in this study.

**Gene**	**Transcript reference**	**qRT-PCR Fw (5'-3')**	**qRT-PCR Rv (5'-3')**	Ref.
vegfaa	ENSDART00000167719	TGCTCCTGCAAATT CACACAA	ATCTTGGCTTTT CACATCTGCAA	Li *et al.* 2012
cxcr4b	ENSDART00000061499	GCGACCTCTCAGT CAGCAAT	TCACAAGCACC ACAAGTCCA	-
cxcl12a	ENSDART00000053946	GCTGGTGCCGTTC CACAGTCA	GGGGCAGTTGG GTGTGTGGAG	-
tnfa	ENSDART00000025847	AGACCTTAGACTG GAGAGATGAC	CAAAGACACCT GGCTGTAGAC	Stockhammer *et al.* 2009
cxcl11aa	ENSDART00000169606	ACTCAACATGGTG AAGCCAGTGCT	CTTCAGCGTGGC TATGACTTCCAT	Torraca *et al.* 2015
cxcl18b	ENSDART00000111598	TCTTCTGCTGCTGC TTGCGGT	GGTGTCCCTGCG AGCACGAT	Van der Vaart *et al.* 2013
il1b	ENSDART00000169225	GAACAGAATGAAG CACATCAAACC	ACGGCACTGAAT CCACCAC	Stockhammer *et al.* 2009
mmp9	ENSDART00000062845	CATTAAAGATGCCC TGATGTATCCC	AGTGGTGGTCCG TGGTTGAG	Stockhammer *et al.* 2009
ppiab	ENSDART00000166085	ACACTGAAACACG GAGGCAAAG	CATCCACAACCT TCCCGAACAC	Stockhammer *et al.* 2009
eif4a1b	ENSDART00000140602 ENSDART00000011878	TTCAGAAACTCAG TACTAGCATACA	GTGACATCCAAC ACCTCTGC	Benard *et al.* 2015
